# Crystal structure of *N*,*N*-diisopropyl-4-methyl­benzene­sulfonamide

**DOI:** 10.1107/S2056989020007185

**Published:** 2020-06-05

**Authors:** Brock A. Stenfors, Richard J. Staples, Shannon M. Biros, Felix N. Ngassa

**Affiliations:** a Department of Chemistry, Grand Valley State University, 1 Campus Dr., Allendale, MI 49401, USA; b Center for Crystallographic Research, Department of Chemistry, Michigan State University, East Lansing, MI 48824, USA

**Keywords:** crystal structure, sulfonamide, intra­molecular C—H⋯O hydrogen bond, inter­molecular C—H⋯O hydrogen bond, inter­molecular C—H⋯π inter­action

## Abstract

The synthesis of the title compound, C_13_H_21_NO_2_S, is reported here along with its crystal structure. This compound crystallizes with two mol­ecules in the asymmetric unit. The sulfonamide functional group of this structure features S=O bond lengths ranging from 1.433 (3) to 1.439 (3) Å, S—C bond lengths of 1.777 (3) and 1.773 (4) Å, and S—N bond lengths of 1.622 (3) and 1.624 (3) Å. When viewing the mol­ecules down the S—N bond, the isopropyl groups are *gauche* to the aromatic ring. On each mol­ecule, two methyl hydrogen atoms of one isopropyl group are engaged in intra­molecular C—H⋯O hydrogen bonds with a nearby sulfonamide oxygen atom. Inter­molecular C—H⋯O hydrogen bonds and C—H⋯π inter­actions link mol­ecules of the title compound in the solid state.

## Abstract 2

The synthesis and crystal structure of a diisopropyl-substituted *p*-tolunesulfonamide is discussed. This structure features C—H⋯O hydrogen bonds (both intra- and inter­molecular) and inter­molecular C—H⋯π inter­actions.

## Chemical context   

Sulfonamides are biologically significant compounds that were first introduced as potent anti­bacterial agents (Chohan *et al.*, 2005 ▸). Since then, sulfonamides have been reported to exhibit a variety of therapeutic properties. These properties include the inhibition of hepatitis C virus (HCV). First discovered in 1989, HCV is a liver disease that is responsible for the majority of liver-related deaths (Chen & Morgan, 2006 ▸; Morozov & Lagaye, 2018 ▸). According to data published in 2016, approximately 69.6 million individuals are affected by HCV (Hill *et al.*, 2017 ▸).

**Figure 1:**
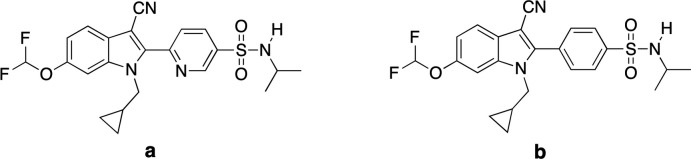
Compounds structurally similar to the title compound, (*a*) 6-(indol-2-yl)pyridine-3-sulfonamides and (*b*) 4-(indol-2-yl)benzene sulfonamides, reported to inhibit hepatitis C virus NS4B.

Advances in HCV treatment have brought about a variety of novel HCV inhibitors that contain the sulfonamide moiety (Johansson *et al.*, 2003 ▸; Gopalsamy *et al.*, 2006 ▸). The pan genotypic NS5A inhibitor, Daclatasvir, and the NS3/4A protease inhibitor, Simeprevir, are examples of sulfonamide drugs approved for the treatment of HCV (Zeuzem *et al.*, 2016 ▸). Aryl­sulfonamides, similar in structure to the title compound, were discovered as potent hepatitis C virus (HCV) 1b replicon inhibitors that target the HCV NS4B protein (Fig. 1 ▸; Zhang *et al.*, 2013 ▸). The HCV NS4B protein is a key component for the replication of HCV RNA (Blight, 2011 ▸). It is necessary to synthesize a variety of potential inhibitors to work towards the treatment of HCV.

Producing biologically significant sulfonamide compounds is highly dependent on a facile synthetic method. A review of the current literature suggests a viable route of synthesis is the reaction between an electrophilic sulfonyl chloride and nucleophilic amine (Almarhoon *et al.*, 2019 ▸). Another notable method for synthesizing sulfonamide compounds is the reaction between an *N*-silyl­amine and a sulfonyl chloride (Naredla & Klumpp, 2013 ▸). The title compound was synthesized by an analogous nucleophilic acyl substitution reaction between *p*-toluene­sulfonyl chloride and diiso­propyl­amine in the presence of pyridine. The use of *p*-toluene­sulfonyl chloride as an electrophile is a good starting point in synthesizing aryl­sulfonamides due to its availability and low cost. Herein, we report the synthesis and crystal structure of *N*,*N*-diisopropyl-4-methyl­benzene­sulfonamide. The crystal structure of the title compound was obtained *via* single crystal X-ray diffraction.
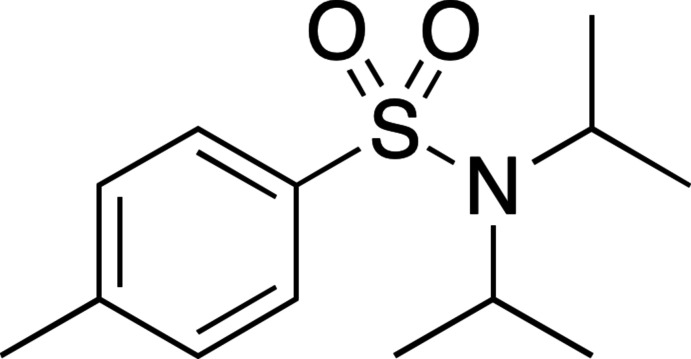


## Structural commentary   

**Figure 2:**
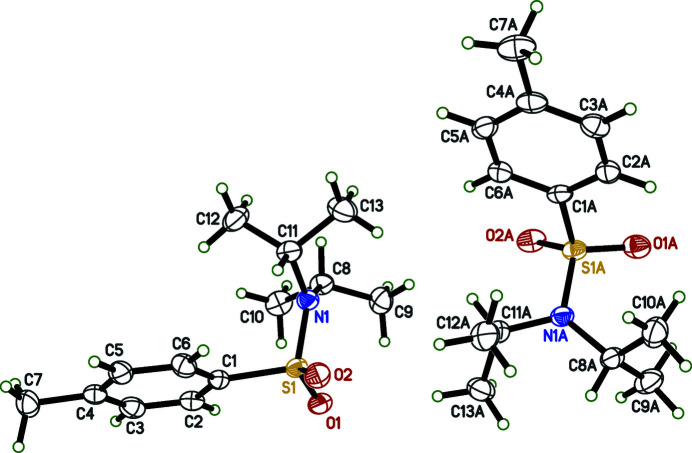
The mol­ecular structure of the title compound, with the atom labeling scheme for both crystallographically unique mol­ecules. Displacement ellipsoids are shown at the 40% probability level using standard CPK colors, and all hydrogen atoms have been omitted for clarity.

**Figure 3:**
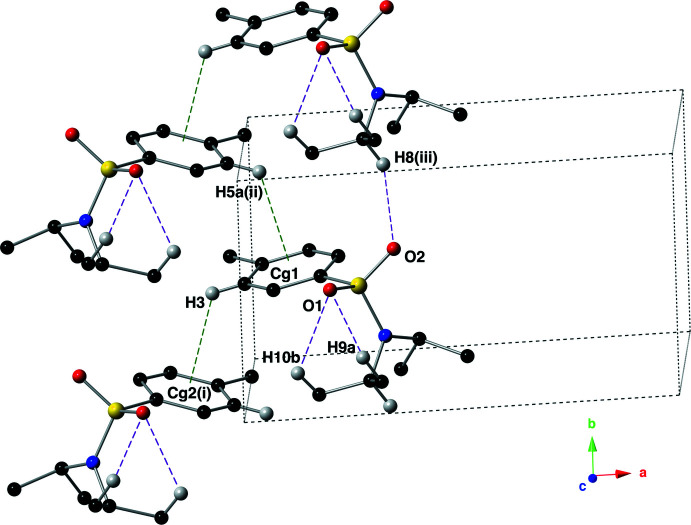
Non-covalent inter­actions present in the title compound, using a ball and stick model with standard CPK colors. C—H⋯π inter­actions are drawn with green, dashed lines and C—H⋯O hydrogen bonds are drawn with purple, dashed lines. Symmetry codes: (i) −1 + *x*, −*y*, −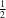 + *z*; (ii) 1 + *x*, 1 − *y*, 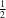 + *z*; (iii) *x*, −1 + *y*, *z*.

The title compound crystallizes in the monoclinic space group *Pc*, with two equivalents of the mol­ecule in the asymmetric unit. The structure was solved with a Flack parameter of 0.002 (14) (Parsons *et al.*, 2013 ▸). The atom labeling scheme is shown in Fig. 2 ▸. The mol­ecules boast S=O bond lengths ranging from 1.433 (3) to 1.439 (3) Å, S—N bond lengths of 1.622 (3) and 1.624 (3) Å, and S—C bond lengths of 1.777 (3) and 1.773 (4) Å. These values lie in the expected ranges for an aromatic sulfonamide group. The O—S—O bond angles for each mol­ecule are 119.35 (16) and 119.54 (16)°. When the mol­ecules are viewed down the S—N bond, both have adopted a similar conformation with the isopropyl groups being *gauche* to the aromatic ring. In each mol­ecule, the methine carbon atom of one of the isopropyl groups is nearly coplanar with a sulfonamide oxygen with O1—S1—N1—C8 and O1*A*—S1*A*—N1*A*—C8*A* torsion angles of 17.1 (3) and 15.7 (3)°, respectively. We attribute this relatively small torsion angle to the presence of intra­molecular C-H⋯O inter­actions, which are described in more detail below. The torsion angles (O2—S1—N1—C11 and O2*A*—S1*A*—N1*A*—C11*A*) between the methine carbon atom of the other isopropyl group and the other sulfonamide oxygen are 46.7 (3) and 46.8 (3)°, respectively. Both sulfur atoms adopt a slightly distorted tetra­hedral geometry with τ4 descriptors for fourfold coordination of 0.94 for both S1 and S1*A* (where 0 = square planar, 0.85 = trigonal pyramidal, and 1 = tetra­hedral; Yang *et al.*, 2007 ▸). Finally, there are two intra­molecular C—H⋯O hydrogen bonds present between one sulfonamide oxygen atom and the methyl hydrogen atoms of an adjacent isopropyl group (Sutor, 1958 ▸, 1962 ▸, 1963 ▸; Steiner, 1996 ▸). While these inter­actions could be simply due to sterics since the *D*—H⋯*A* angles are around 120° (see below), we describe them here as potential C—H⋯O hydrogen bonds. Specifically, O1 inter­acts with C9(H9*A*) and C10(H10*B*), while the equivalent atom O1a inter­acts with C9*A*(H9*AC*) and C10*A*(H10*F*). These inter­actions have *D*⋯*A* distances ranging from 3.039 (5) to 3.157 (5) Å and *D*—H⋯*A* angles ranging from 117 to 121° (Table 1 ▸, Fig. 3 ▸).

## Supra­molecular features   

**Figure 4:**
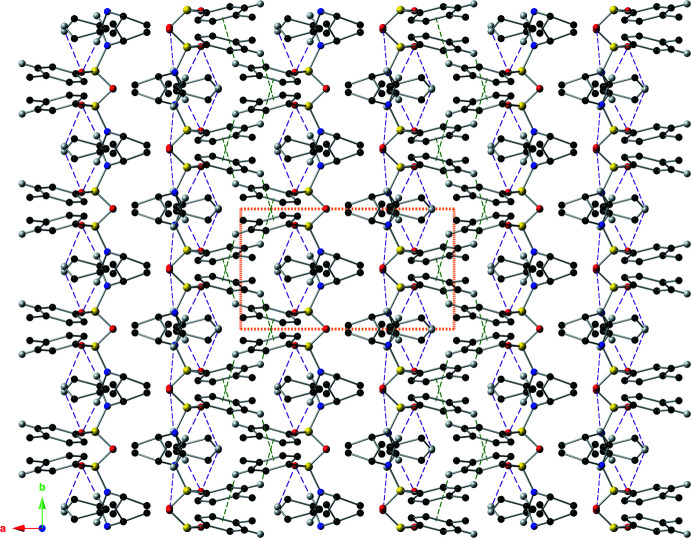
Supra­molecular ribbons of the title compound assembled *via* inter­molecular C—H⋯π inter­actions and C—H⋯O hydrogen bonds, as viewed down the *c* axis using a ball-and-stick model with standard CPK colors. The non-covalent inter­actions are depicted with dashed lines (C—H⋯π: green; C—H⋯O hydrogen bonds: purple), and the unit cell is drawn with orange. Only hydrogen atoms involved in a non-covalent inter­action are shown for clarity.

Mol­ecules of the title compound are held together in the solid state by inter­molecular C–H⋯π inter­actions and C–H⋯O hydrogen bonds (Fig. 3 ▸). The C–H⋯π inter­actions have C⋯centroid distances of 3.515 (4) and 3.548 (4) Å, with C—H··centroid angles of 120 and 121°. The inter­molecular C—H⋯O hydrogen bond is present between C8(H8) and O2^i^ [symmetry code: (i) *x*, −1 + *y*, *z*] with a *D*⋯*A* distance of 3.465 (4) Å and a *D*—H⋯*A* angle of 150.8° (Table 1 ▸). These supra­molecular inter­actions form ribbons that run parallel to the *b*-axis direction (Fig. 4 ▸).

## Database survey   

A search of the Cambridge Structural Database (CSD, Version 5.41, November, 2019; Groom *et al.*, 2016 ▸) reveals over 5,000 structures of *p*-methyl­benzene­sulfonamides where the nitro­gen atom bears two carbon groups. A few structures that have relatively simple –*R* groups bonded to the sulfonamide nitro­gen atom are RUGQEQ (Khan *et al.*, 2009 ▸), CIQGOZ (Zhou & Zheng, 2007 ▸) and CEMFUX (Zhou *et al.*, 2012 ▸). In the structure of RUGQEQ, the sulfonamide nitro­gen atom bears a benzyl and a cyclo­hexyl group, while in CIQGOZ the –*R* groups are methyl and phenyl. For the structure CEMFUX, two sulfonamide nitro­gen atoms are linked *via* an ethyl­ene chain, and the other –*R* group is a substituted propyl ester.

## Synthesis and crystallization   

The title compound was prepared by the dropwise addition of *p*-toluene­sulfonyl chloride (1.00 g, 5.25 mmol) to a stirring mixture of diiso­propyl­amine (0.83 mL, 5.90 mmol), pyridine (0.48 mL, 5.90 mmol) and 10 mL of degassed di­chloro­methane under a nitro­gen atmosphere. The reaction mixture was stirred at room temperature for 24 h under a nitro­gen atmosphere. After acidification with 5 *M* HCl and dilution with 15 mL of di­chloro­methane, the organic layer was washed with water and brine. The aqueous layers were back extracted with 10 mL of di­chloro­methane. The combined organic layers were then dried over anhydrous sodium sulfate and evaporated to dryness. The resulting solid was dissolved in hot ethanol and filtered. The filtrate was placed in a freezer for two days and the product was isolated *via* vacuum filtration to give colorless crystals (13%; m.p. 362–365 K).

## Refinement   

Crystal data, data collection and structure refinement details are summarized in Table 2 ▸. For this structure, hydrogen atoms bonded to carbon atoms were placed in calculated positions and refined as riding: C—H = 0.95–1.00 Å with *U*_iso_(H) = 1.2*U*_eq_(C) for methine groups and aromatic hydrogen atoms, and *U*_iso_(H) = 1.5*U*_eq_(C) for methyl groups.

## Supplementary Material

Crystal structure: contains datablock(s) I. DOI: 10.1107/S2056989020007185/pk2628sup1.cif

Structure factors: contains datablock(s) I. DOI: 10.1107/S2056989020007185/pk2628Isup2.hkl

Supporting information file. DOI: 10.1107/S2056989020007185/pk2628Isup3.cml

CCDC reference: 2006237

Additional supporting information:  crystallographic information; 3D view; checkCIF report

## Figures and Tables

**Table 1:** Hydrogen-bond geometry (Å, °) *Cg*1 and *Cg*2 are the centroids of the C1–C6 and C1*A*–C6*A* rings, respectively.

*D*—H⋯*A*	*D*—H	H⋯*A*	*D*⋯*A*	*D*—H⋯*A*
C8—H8⋯O2^i^	1.00	2.56	3.464 (4)	151
C9—H9*A*⋯O1	0.98	2.44	3.071 (5)	121
C10—H10*B*⋯O1	0.98	2.59	3.157 (5)	117
C9*A*—H9*AC*⋯O1*A*	0.98	2.41	3.039 (5)	121
C10*A*—H10*F*⋯O1*A*	0.98	2.57	3.157 (5)	118
C3—H3⋯*Cg*2^ii^	0.95	2.95	3.515 (4)	120
C3*A*—H3*A*⋯*Cg*1^iii^	0.95	2.96	3.548 (4)	121

Symmetry codes: (i) 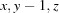; (ii) 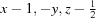; (iii) 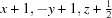.

**Table 2:** Experimental details

Crystal data	
Chemical formula	C_13_H_21_NO_2_S
*M*_r_	255.37
Crystal system, space group	Monoclinic, *P* *c*
Temperature (K)	173
*a*, *b*, *c* (Å)	12.87828 (18), 6.88418 (10), 16.2080 (2)
β (°)	108.1513 (8)
*V* (Å^3^)	1365.43 (3)
*Z*	4
Radiation type	Cu *K*α
μ (mm^−1^)	2.03
Crystal size (mm)	0.18 × 0.16 × 0.14

Data collection	
Diffractometer	Bruker APEXII CCD
Absorption correction	Multi-scan (*SADABS*; Bruker, 2013 ▸)
*T*_min_, *T*_max_	0.640, 0.754
No. of measured, independent and observed [*I* > 2σ(*I*)] reflections	15150, 4924, 4746
*R*_int_	0.034
(sin θ/λ)_max_ (Å^−1^)	0.617

Refinement	
*R*[*F* ^2^ > 2σ(*F* ^2^)], *wR*(*F* ^2^), *S*	0.042, 0.109, 1.07
No. of reflections	4924
No. of parameters	317
No. of restraints	2
H-atom treatment	H-atom parameters constrained
Δρ_max_, Δρ_min_ (e Å^−3^)	0.63, −0.24
Absolute structure	Flack *x* determined using 2083 quotients [(*I* ^+^)−(*I* ^−^)]/[(*I* ^+^)+(*I* ^−^)] (Parsons *et al.*, 2013 ▸)
Absolute structure parameter	0.002 (14)

Computer programs: *APEX2* and *SAINT* (Bruker, 2013 ▸), *SHELXS* (Sheldrick, 2008 ▸), *SHELXL* (Sheldrick, 2015 ▸), *OLEX2* (Dolomanov *et al.*, 2009 ▸; Bourhis *et al.*, 2015 ▸), *XP* (Sheldrick, 2008 ▸) and *CrystalMaker* (Palmer, 2007 ▸).

## supplementary crystallographic information

### Crystal data

**Table:**

C_13_H_21_NO_2_S	*F*(000) = 552
*M**_r_* = 255.37	*D*_x_ = 1.242 Mg m^−^^3^
Monoclinic, *P**c*	Cu *K*α radiation, λ = 1.54178 Å
*a* = 12.87828 (18) Å	Cell parameters from 9971 reflections
*b* = 6.88418 (10) Å	θ = 3.6–72.1°
*c* = 16.2080 (2) Å	µ = 2.03 mm^−^^1^
β = 108.1513 (8)°	*T* = 173 K
*V* = 1365.43 (3) Å^3^	Block, colourless
*Z* = 4	0.18 × 0.16 × 0.14 mm

### Data collection

**Table:**

Bruker APEXII CCD diffractometer	4746 reflections with *I* > 2σ(*I*)
φ and ω scans	*R*_int_ = 0.034
Absorption correction: multi-scan (SADABS; Bruker, 2013)	θ_max_ = 72.1°, θ_min_ = 3.6°
*T*_min_ = 0.640, *T*_max_ = 0.754	*h* = −15→15
15150 measured reflections	*k* = −8→8
4924 independent reflections	*l* = −20→18

### Refinement

**Table:**

Refinement on *F*^2^	Hydrogen site location: inferred from neighbouring sites
Least-squares matrix: full	H-atom parameters constrained
*R*[*F*^2^ > 2σ(*F*^2^)] = 0.042	*w* = 1/[σ^2^(*F*_o_^2^) + (0.0814*P*)^2^ + 0.0782*P*] where *P* = (*F*_o_^2^ + 2*F*_c_^2^)/3
*wR*(*F*^2^) = 0.109	(Δ/σ)_max_ < 0.001
*S* = 1.07	Δρ_max_ = 0.63 e Å^−^^3^
4924 reflections	Δρ_min_ = −0.24 e Å^−^^3^
317 parameters	Absolute structure: Flack *x* determined using 2083 quotients [(*I*^+^)-(*I*^-^)]/[(*I*^+^)+(*I*^-^)] (Parsons *et al.*, 2013)
2 restraints	Absolute structure parameter: 0.002 (14)
Primary atom site location: structure-invariant direct methods	

### Special details

**Table:**

Geometry. All esds (except the esd in the dihedral angle between two l.s. planes) are estimated using the full covariance matrix. The cell esds are taken into account individually in the estimation of esds in distances, angles and torsion angles; correlations between esds in cell parameters are only used when they are defined by crystal symmetry. An approximate (isotropic) treatment of cell esds is used for estimating esds involving l.s. planes.

### Fractional atomic coordinates and isotropic or equivalent isotropic displacement parameters (Å^2^)

**Table:**

	*x*	*y*	*z*	*U*_iso_*/*U*_eq_	
S1	0.25085 (5)	0.34566 (10)	0.27005 (5)	0.0236 (2)	
O1	0.1864 (2)	0.3511 (4)	0.32791 (17)	0.0323 (6)	
O2	0.3339 (2)	0.4906 (3)	0.27789 (17)	0.0315 (5)	
N1	0.3086 (2)	0.1339 (4)	0.27951 (19)	0.0249 (6)	
C1	0.1606 (3)	0.3671 (5)	0.1623 (2)	0.0240 (7)	
C2	0.0507 (3)	0.3170 (5)	0.1436 (2)	0.0274 (7)	
H2	0.0228	0.2759	0.1885	0.033*	
C3	−0.0170 (3)	0.3280 (5)	0.0586 (3)	0.0308 (7)	
H3	−0.0918	0.2937	0.0456	0.037*	
C4	0.0219 (3)	0.3880 (5)	−0.0081 (2)	0.0298 (7)	
C5	0.1322 (3)	0.4381 (5)	0.0119 (2)	0.0303 (7)	
H5	0.1603	0.4787	−0.0330	0.036*	
C6	0.2006 (3)	0.4289 (5)	0.0968 (2)	0.0291 (7)	
H6	0.2752	0.4652	0.1101	0.035*	
C7	−0.0533 (3)	0.4041 (6)	−0.1000 (3)	0.0393 (9)	
H7A	−0.1056	0.5097	−0.1038	0.059*	
H7B	−0.0930	0.2816	−0.1171	0.059*	
H7C	−0.0103	0.4315	−0.1390	0.059*	
C8	0.2723 (3)	−0.0367 (5)	0.3194 (2)	0.0271 (7)	
H8	0.3160	−0.1489	0.3092	0.033*	
C9	0.3011 (3)	−0.0184 (6)	0.4176 (3)	0.0365 (8)	
H9A	0.2604	0.0900	0.4318	0.055*	
H9B	0.3797	0.0054	0.4428	0.055*	
H9C	0.2818	−0.1391	0.4415	0.055*	
C10	0.1530 (3)	−0.0920 (6)	0.2761 (3)	0.0357 (8)	
H10A	0.1378	−0.0968	0.2130	0.054*	
H10B	0.1056	0.0050	0.2905	0.054*	
H10C	0.1389	−0.2198	0.2971	0.054*	
C11	0.3851 (3)	0.0994 (5)	0.2283 (2)	0.0279 (7)	
H11	0.3971	0.2268	0.2030	0.034*	
C12	0.3388 (4)	−0.0400 (6)	0.1535 (3)	0.0396 (9)	
H12A	0.2677	0.0079	0.1168	0.059*	
H12B	0.3296	−0.1685	0.1763	0.059*	
H12C	0.3890	−0.0495	0.1190	0.059*	
C13	0.4957 (3)	0.0315 (7)	0.2889 (3)	0.0405 (9)	
H13A	0.4871	−0.0953	0.3135	0.061*	
H13B	0.5230	0.1260	0.3359	0.061*	
H13C	0.5478	0.0203	0.2561	0.061*	
S1A	0.68214 (5)	0.14554 (10)	0.62704 (5)	0.0245 (2)	
O1A	0.7462 (2)	0.1368 (4)	0.71678 (18)	0.0341 (6)	
O2A	0.5988 (2)	0.0023 (4)	0.59227 (18)	0.0321 (6)	
N1A	0.6249 (2)	0.3582 (4)	0.60873 (19)	0.0245 (6)	
C1A	0.7733 (3)	0.1253 (5)	0.5650 (2)	0.0244 (6)	
C2A	0.8822 (3)	0.1807 (5)	0.6000 (2)	0.0273 (7)	
H2A	0.9094	0.2242	0.6584	0.033*	
C3A	0.9505 (3)	0.1721 (5)	0.5491 (3)	0.0298 (7)	
H3A	1.0248	0.2099	0.5732	0.036*	
C4A	0.9125 (3)	0.1091 (5)	0.4631 (2)	0.0295 (7)	
C5A	0.8030 (3)	0.0539 (5)	0.4289 (2)	0.0300 (7)	
H5A	0.7754	0.0116	0.3703	0.036*	
C6A	0.7346 (3)	0.0606 (5)	0.4797 (2)	0.0275 (7)	
H6A	0.6606	0.0207	0.4561	0.033*	
C7A	0.9887 (3)	0.0957 (6)	0.4096 (3)	0.0395 (9)	
H7AA	1.0400	−0.0115	0.4310	0.059*	
H7AB	1.0293	0.2177	0.4142	0.059*	
H7AC	0.9464	0.0723	0.3488	0.059*	
C8A	0.6606 (3)	0.5254 (5)	0.6691 (2)	0.0284 (7)	
H8A	0.6168	0.6393	0.6390	0.034*	
C9A	0.6315 (4)	0.4982 (6)	0.7524 (3)	0.0400 (9)	
H9AA	0.5536	0.4672	0.7381	0.060*	
H9AB	0.6472	0.6181	0.7867	0.060*	
H9AC	0.6749	0.3916	0.7863	0.060*	
C10A	0.7800 (3)	0.5825 (6)	0.6868 (3)	0.0396 (9)	
H10E	0.7948	0.7034	0.7204	0.059*	
H10D	0.7948	0.6015	0.6316	0.059*	
H10F	0.8273	0.4791	0.7199	0.059*	
C11A	0.5500 (3)	0.3973 (5)	0.5205 (2)	0.0285 (7)	
H11A	0.5371	0.2713	0.4882	0.034*	
C12A	0.5984 (3)	0.5382 (6)	0.4700 (3)	0.0382 (8)	
H12D	0.6099	0.6647	0.4992	0.057*	
H12E	0.5480	0.5529	0.4110	0.057*	
H12F	0.6684	0.4876	0.4674	0.057*	
C13A	0.4399 (3)	0.4693 (6)	0.5249 (3)	0.0371 (8)	
H13D	0.4116	0.3783	0.5592	0.056*	
H13E	0.3883	0.4781	0.4660	0.056*	
H13F	0.4489	0.5978	0.5522	0.056*	

### Atomic displacement parameters (Å^2^)

**Table:**

	*U*^11^	*U*^22^	*U*^33^	*U*^12^	*U*^13^	*U*^23^
S1	0.0291 (4)	0.0182 (4)	0.0235 (4)	0.0012 (3)	0.0081 (3)	−0.0022 (3)
O1	0.0406 (14)	0.0291 (14)	0.0286 (13)	0.0074 (10)	0.0126 (10)	−0.0021 (9)
O2	0.0357 (12)	0.0214 (11)	0.0341 (13)	−0.0035 (10)	0.0062 (10)	−0.0020 (9)
N1	0.0300 (15)	0.0225 (14)	0.0233 (15)	0.0042 (11)	0.0100 (12)	0.0020 (10)
C1	0.0288 (15)	0.0201 (15)	0.0230 (16)	0.0045 (12)	0.0080 (12)	0.0001 (11)
C2	0.0284 (15)	0.0218 (14)	0.0338 (17)	0.0031 (13)	0.0123 (13)	0.0005 (12)
C3	0.0286 (15)	0.0212 (15)	0.0400 (19)	0.0001 (12)	0.0070 (14)	−0.0023 (13)
C4	0.0387 (17)	0.0178 (14)	0.0301 (18)	0.0067 (14)	0.0067 (14)	−0.0014 (12)
C5	0.0381 (17)	0.0265 (17)	0.0280 (17)	0.0061 (14)	0.0130 (13)	0.0056 (13)
C6	0.0300 (15)	0.0265 (17)	0.0313 (18)	0.0021 (13)	0.0104 (13)	0.0052 (13)
C7	0.049 (2)	0.0281 (18)	0.0316 (19)	0.0017 (16)	−0.0012 (15)	−0.0011 (14)
C8	0.0343 (16)	0.0201 (15)	0.0298 (17)	0.0005 (13)	0.0140 (13)	0.0030 (13)
C9	0.047 (2)	0.0337 (19)	0.0285 (18)	−0.0011 (16)	0.0117 (15)	0.0063 (14)
C10	0.0382 (18)	0.0300 (17)	0.043 (2)	−0.0069 (15)	0.0182 (15)	−0.0011 (15)
C11	0.0323 (16)	0.0247 (15)	0.0301 (17)	0.0029 (13)	0.0144 (13)	0.0013 (13)
C12	0.052 (2)	0.040 (2)	0.0306 (19)	0.0053 (18)	0.0188 (16)	−0.0065 (15)
C13	0.0327 (17)	0.046 (2)	0.045 (2)	0.0097 (16)	0.0149 (16)	0.0061 (17)
S1A	0.0310 (4)	0.0190 (4)	0.0267 (4)	0.0036 (3)	0.0135 (3)	0.0040 (3)
O1A	0.0438 (15)	0.0305 (13)	0.0311 (14)	0.0098 (11)	0.0161 (11)	0.0078 (9)
O2A	0.0371 (12)	0.0230 (12)	0.0422 (15)	−0.0022 (10)	0.0211 (11)	0.0008 (10)
N1A	0.0289 (14)	0.0197 (13)	0.0256 (15)	0.0031 (10)	0.0096 (11)	−0.0003 (10)
C1A	0.0304 (16)	0.0191 (14)	0.0274 (17)	0.0070 (12)	0.0141 (13)	0.0023 (11)
C2A	0.0284 (15)	0.0231 (15)	0.0296 (17)	0.0022 (13)	0.0078 (13)	0.0000 (12)
C3A	0.0286 (15)	0.0214 (15)	0.0398 (19)	0.0018 (13)	0.0114 (13)	0.0035 (13)
C4A	0.0370 (17)	0.0182 (14)	0.0381 (19)	0.0080 (13)	0.0185 (15)	0.0069 (13)
C5A	0.0396 (17)	0.0248 (16)	0.0269 (17)	0.0072 (14)	0.0120 (13)	−0.0004 (12)
C6A	0.0274 (14)	0.0242 (16)	0.0300 (17)	0.0051 (13)	0.0075 (12)	−0.0001 (13)
C7A	0.047 (2)	0.0306 (18)	0.052 (2)	0.0040 (16)	0.0325 (18)	0.0047 (16)
C8A	0.0350 (16)	0.0237 (15)	0.0260 (16)	0.0044 (13)	0.0089 (13)	−0.0022 (12)
C9A	0.055 (2)	0.038 (2)	0.0320 (19)	0.0065 (18)	0.0203 (17)	−0.0040 (15)
C10A	0.042 (2)	0.038 (2)	0.035 (2)	−0.0047 (17)	0.0059 (15)	−0.0056 (16)
C11A	0.0335 (17)	0.0251 (16)	0.0254 (16)	0.0041 (14)	0.0070 (13)	−0.0006 (13)
C12A	0.051 (2)	0.039 (2)	0.0285 (18)	0.0049 (17)	0.0186 (16)	0.0072 (15)
C13A	0.0298 (16)	0.0352 (19)	0.045 (2)	0.0068 (15)	0.0091 (14)	0.0003 (16)

### Geometric parameters (Å, º)

**Table:**

S1—O1	1.434 (3)	S1A—O1A	1.433 (3)
S1—O2	1.439 (3)	S1A—O2A	1.437 (3)
S1—N1	1.622 (3)	S1A—N1A	1.624 (3)
S1—C1	1.777 (3)	S1A—C1A	1.773 (4)
N1—C8	1.484 (4)	N1A—C8A	1.489 (4)
N1—C11	1.493 (4)	N1A—C11A	1.479 (4)
C1—C2	1.395 (5)	C1A—C2A	1.393 (5)
C1—C6	1.383 (5)	C1A—C6A	1.390 (5)
C2—H2	0.9500	C2A—H2A	0.9500
C2—C3	1.384 (5)	C2A—C3A	1.381 (5)
C3—H3	0.9500	C3A—H3A	0.9500
C3—C4	1.388 (6)	C3A—C4A	1.395 (5)
C4—C5	1.398 (5)	C4A—C5A	1.398 (5)
C4—C7	1.507 (5)	C4A—C7A	1.500 (5)
C5—H5	0.9500	C5A—H5A	0.9500
C5—C6	1.385 (5)	C5A—C6A	1.381 (5)
C6—H6	0.9500	C6A—H6A	0.9500
C7—H7A	0.9800	C7A—H7AA	0.9800
C7—H7B	0.9800	C7A—H7AB	0.9800
C7—H7C	0.9800	C7A—H7AC	0.9800
C8—H8	1.0000	C8A—H8A	1.0000
C8—C9	1.524 (5)	C8A—C9A	1.521 (5)
C8—C10	1.525 (5)	C8A—C10A	1.525 (5)
C9—H9A	0.9800	C9A—H9AA	0.9800
C9—H9B	0.9800	C9A—H9AB	0.9800
C9—H9C	0.9800	C9A—H9AC	0.9800
C10—H10A	0.9800	C10A—H10D	0.9800
C10—H10B	0.9800	C10A—H10E	0.9800
C10—H10C	0.9800	C10A—H10F	0.9800
C11—H11	1.0000	C11A—H11A	1.0000
C11—C12	1.516 (5)	C11A—C12A	1.522 (5)
C11—C13	1.529 (5)	C11A—C13A	1.525 (5)
C12—H12A	0.9800	C12A—H12D	0.9800
C12—H12B	0.9800	C12A—H12E	0.9800
C12—H12C	0.9800	C12A—H12F	0.9800
C13—H13A	0.9800	C13A—H13D	0.9800
C13—H13B	0.9800	C13A—H13E	0.9800
C13—H13C	0.9800	C13A—H13F	0.9800
			
O1—S1—O2	119.35 (16)	O1A—S1A—O2A	119.54 (16)
O1—S1—N1	107.65 (15)	O1A—S1A—N1A	107.96 (15)
O1—S1—C1	107.80 (16)	O1A—S1A—C1A	107.38 (17)
O2—S1—N1	107.99 (14)	O2A—S1A—N1A	107.79 (15)
O2—S1—C1	105.66 (16)	O2A—S1A—C1A	105.62 (16)
N1—S1—C1	107.92 (15)	N1A—S1A—C1A	108.09 (15)
C8—N1—S1	123.7 (2)	C8A—N1A—S1A	123.2 (2)
C8—N1—C11	117.9 (3)	C11A—N1A—S1A	117.6 (2)
C11—N1—S1	117.0 (2)	C11A—N1A—C8A	118.1 (3)
C2—C1—S1	120.1 (3)	C2A—C1A—S1A	120.6 (3)
C6—C1—S1	119.6 (3)	C6A—C1A—S1A	119.6 (3)
C6—C1—C2	120.3 (3)	C6A—C1A—C2A	119.8 (3)
C1—C2—H2	120.5	C1A—C2A—H2A	120.2
C3—C2—C1	119.0 (3)	C3A—C2A—C1A	119.6 (3)
C3—C2—H2	120.5	C3A—C2A—H2A	120.2
C2—C3—H3	119.2	C2A—C3A—H3A	119.3
C2—C3—C4	121.6 (3)	C2A—C3A—C4A	121.3 (3)
C4—C3—H3	119.2	C4A—C3A—H3A	119.3
C3—C4—C5	118.6 (3)	C3A—C4A—C5A	118.5 (3)
C3—C4—C7	121.0 (3)	C3A—C4A—C7A	120.5 (3)
C5—C4—C7	120.3 (3)	C5A—C4A—C7A	121.0 (3)
C4—C5—H5	119.8	C4A—C5A—H5A	119.8
C6—C5—C4	120.3 (3)	C6A—C5A—C4A	120.5 (3)
C6—C5—H5	119.8	C6A—C5A—H5A	119.8
C1—C6—C5	120.2 (3)	C1A—C6A—H6A	119.8
C1—C6—H6	119.9	C5A—C6A—C1A	120.4 (3)
C5—C6—H6	119.9	C5A—C6A—H6A	119.8
C4—C7—H7A	109.5	C4A—C7A—H7AA	109.5
C4—C7—H7B	109.5	C4A—C7A—H7AB	109.5
C4—C7—H7C	109.5	C4A—C7A—H7AC	109.5
H7A—C7—H7B	109.5	H7AA—C7A—H7AB	109.5
H7A—C7—H7C	109.5	H7AA—C7A—H7AC	109.5
H7B—C7—H7C	109.5	H7AB—C7A—H7AC	109.5
N1—C8—H8	105.7	N1A—C8A—H8A	105.8
N1—C8—C9	112.5 (3)	N1A—C8A—C9A	112.1 (3)
N1—C8—C10	114.0 (3)	N1A—C8A—C10A	114.2 (3)
C9—C8—H8	105.7	C9A—C8A—H8A	105.8
C9—C8—C10	112.5 (3)	C9A—C8A—C10A	112.2 (3)
C10—C8—H8	105.7	C10A—C8A—H8A	105.8
C8—C9—H9A	109.5	C8A—C9A—H9AA	109.5
C8—C9—H9B	109.5	C8A—C9A—H9AB	109.5
C8—C9—H9C	109.5	C8A—C9A—H9AC	109.5
H9A—C9—H9B	109.5	H9AA—C9A—H9AB	109.5
H9A—C9—H9C	109.5	H9AA—C9A—H9AC	109.5
H9B—C9—H9C	109.5	H9AB—C9A—H9AC	109.5
C8—C10—H10A	109.5	C8A—C10A—H10D	109.5
C8—C10—H10B	109.5	C8A—C10A—H10E	109.5
C8—C10—H10C	109.5	C8A—C10A—H10F	109.5
H10A—C10—H10B	109.5	H10D—C10A—H10E	109.5
H10A—C10—H10C	109.5	H10D—C10A—H10F	109.5
H10B—C10—H10C	109.5	H10E—C10A—H10F	109.5
N1—C11—H11	107.5	N1A—C11A—H11A	107.5
N1—C11—C12	112.5 (3)	N1A—C11A—C12A	112.5 (3)
N1—C11—C13	109.6 (3)	N1A—C11A—C13A	110.5 (3)
C12—C11—H11	107.5	C12A—C11A—H11A	107.5
C12—C11—C13	112.1 (3)	C12A—C11A—C13A	111.1 (3)
C13—C11—H11	107.5	C13A—C11A—H11A	107.5
C11—C12—H12A	109.5	C11A—C12A—H12D	109.5
C11—C12—H12B	109.5	C11A—C12A—H12E	109.5
C11—C12—H12C	109.5	C11A—C12A—H12F	109.5
H12A—C12—H12B	109.5	H12D—C12A—H12E	109.5
H12A—C12—H12C	109.5	H12D—C12A—H12F	109.5
H12B—C12—H12C	109.5	H12E—C12A—H12F	109.5
C11—C13—H13A	109.5	C11A—C13A—H13D	109.5
C11—C13—H13B	109.5	C11A—C13A—H13E	109.5
C11—C13—H13C	109.5	C11A—C13A—H13F	109.5
H13A—C13—H13B	109.5	H13D—C13A—H13E	109.5
H13A—C13—H13C	109.5	H13D—C13A—H13F	109.5
H13B—C13—H13C	109.5	H13E—C13A—H13F	109.5

### Hydrogen-bond geometry (Å, º)

*Cg*1 and *Cg*2 are the centroids of the C1–C6 and C1*A*–C6*A* rings, respectively.

**Table:**

*D*—H···*A*	*D*—H	H···*A*	*D*···*A*	*D*—H···*A*
C8—H8···O2^i^	1.00	2.56	3.464 (4)	151
C9—H9*A*···O1	0.98	2.44	3.071 (5)	121
C10—H10*B*···O1	0.98	2.59	3.157 (5)	117
C9*A*—H9*AC*···O1*A*	0.98	2.41	3.039 (5)	121
C10*A*—H10*F*···O1*A*	0.98	2.57	3.157 (5)	118
C3—H3···*Cg*2^ii^	0.95	2.95	3.515 (4)	120
C3*A*—H3*A*···*Cg*1^iii^	0.95	2.96	3.548 (4)	121

Symmetry codes: (i) *x*, *y*−1, *z*; (ii) *x*−1, −*y*, *z*−1/2; (iii) *x*+1, −*y*+1, *z*+1/2.

## References

[bb1] Almarhoon, Z., Soliman, S. M., Ghabbour, H. A. & El-Faham, A. (2019). *Crystals*, **9**, 35.

[bb2] Blight, K. J. (2011). *J. Virol.* **85**, 8158–8171. 10.1128/JVI.00858-11 PMC3147982 21680530

[bb3] Bourhis, L. J., Dolomanov, O. V., Gildea, R. J., Howard, J. A. K. & Puschmann, H. (2015). *Acta Cryst.* A**71**, 59–75. 10.1107/S2053273314022207 PMC4283469 25537389

[bb4] Bruker (2013). *APEX2*, *SAINT* and *SADABS*. Bruker AXS Inc. Madison, Wisconsin, USA.

[bb5] Chen, S. L. & Morgan, T. R. (2006). *Int. J. Med. Sci.* pp. 47–52.

[bb6] Chohan, Z. H., Mahmood-ul-Hassan, Khan, K. M. & Supuran, C. T. (2005). *J. Enzyme Inhib. Med. Chem.* **20**, 183–188. 10.1080/14756360500043257 15968823

[bb7] Dolomanov, O. V., Bourhis, L. J., Gildea, R. J., Howard, J. A. K. & Puschmann, H. (2009). *J. Appl. Cryst.* **42**, 339–341.

[bb8] Gopalsamy, A., Chopra, R., Lim, K., Ciszewski, G., Shi, M., Curran, K. J., Sukits, S. F., Svenson, K., Bard, J., Ellingboe, J. W., Agarwal, A., Krishnamurthy, G., Howe, A. Y. M., Orlowski, M., Feld, B., O’Connell, J. & Mansour, T. S. (2006). *J. Med. Chem.* **49**, 3052–3055. 10.1021/jm060168g 16722622

[bb9] Groom, C. R., Bruno, I. J., Lightfoot, M. P. & Ward, S. C. (2016). *Acta Cryst.* B**72**, 171–179. 10.1107/S2052520616003954 PMC4822653 27048719

[bb10] Hill, A. M., Nath, S. & Simmons, B. (2017). *J. Virus Erad.* **3**, 117–123. 10.1016/S2055-6640(20)30329-0 PMC5518239 28758018

[bb11] Johansson, A., Poliakov, A., Åkerblom, E., Wiklund, K., Lindeberg, G., Winiwarter, S., Danielson, U. H., Samuelsson, B. & Hallberg, A. (2003). *Bioorg. Med. Chem.* **11**, 2551–2568. 10.1016/s0968-0896(03)00179-2 12757723

[bb12] Khan, I. U., Haider, Z., Zia-ur-Rehman, M., Shafiq, M. & Arshad, M. N. (2009). *Acta Cryst.* E**65**, o3109. 10.1107/S1600536809048193 PMC2972048 21578835

[bb13] Morozov, V. A. & Lagaye, S. (2018). *J. Hepatol.* **10**, 186–212. 10.4254/wjh.v10.i2.186 PMC5838439 29527256

[bb14] Naredla, R. R. & Klumpp, D. A. (2013). *Tetrahedron Lett.* **54**, 5945–5947. 10.1016/j.tetlet.2013.08.034 PMC5333644 28260818

[bb15] Palmer, D. (2007). *CrystalMaker.* CrystalMaker Software Ltd, Bicester, England.

[bb16] Parsons, S., Flack, H. D. & Wagner, T. (2013). *Acta Cryst.* B**69**, 249–259. 10.1107/S2052519213010014 PMC3661305 23719469

[bb17] Sheldrick, G. M. (2008). *Acta Cryst.* A**64**, 112–122. 10.1107/S0108767307043930 18156677

[bb18] Sheldrick, G. M. (2015). *Acta Cryst.* C**71**, 3–8.

[bb19] Steiner, T. (1996). *Crystallogr. Rev.* **6**, 1–51.

[bb20] Sutor, D. J. (1958). *Acta Cryst.* **11**, 453–458.

[bb21] Sutor, D. J. (1962). *Nature*, **195**, 68–69.

[bb22] Sutor, D. J. (1963). *J. Chem. Soc.* pp. 1105–1110.

[bb23] Yang, L., Powell, D. R. & Houser, R. P. (2007). *Dalton Trans.* pp. 955–964. 10.1039/b617136b 17308676

[bb24] Zeuzem, S., Hézode, C., Bronowicki, J., Loustaud-Ratti, V., Gea, F., Buti, M., Olveira, A., Banyai, T., Al-Assi, M. T., Petersen, J., Thabut, D., Gadano, A., Pruitt, R., Makara, M., Bourlière, M., Pol, S., Beumont-Mauviel, M., Ouwerkerk-Mahadevan, S., Picchio, G., Bifano, G., McPhee, F., Boparai, N., Cheung, K., Hughes, E. A. & Noviello, S. (2016). *J. Hepatol.* **64**, 292–300. 10.1016/j.jhep.2015.09.024 26453968

[bb25] Zhang, X., Zhang, N., Chen, G., Turpoff, A., Ren, H., Takasugi, J., Morrill, C., Zhu, J., Li, C., Lennox, W., Paget, S., Liu, Y., Almstead, N., Njoroge, F. G., Gu, Z., Komatsu, T., Clausen, V., Espiritu, C., Graci, J., Colacino, J., Lahser, F., Risher, N., Weetall, M., Nomeir, A. & Karp, G. M. (2013). *Bioorg. Med. Chem. Lett.* **23**, 3947–3953. 10.1016/j.bmcl.2013.04.049 23683597

[bb26] Zhou, B. & Zheng, P.-W. (2007). *Acta Cryst.* E**63**, o4727.

[bb27] Zhou, R., Wang, J., Duan, C. & He, Z. (2012). *Org. Lett.* **14**, 6134–6137. 10.1021/ol302696e 23215234

